# KSHV and Human Diseases: Beyond KS, PEL and MCD

**DOI:** 10.3390/microorganisms14030637

**Published:** 2026-03-12

**Authors:** Caroline Grace Firmin, Lu Dai, Zhiqiang Qin

**Affiliations:** Department of Pathology, Winthrop P. Rockefeller Cancer Institute, University of Arkansas for Medical Sciences, 4301 W Markham St, Little Rock, AR 72205, USA; cgfirmin@uams.edu

**Keywords:** KSHV, KICS, diabetes mellitus, malaria, cervical cancer, prostate cancer, heart disease, osteosarcoma

## Abstract

Kaposi’s Sarcoma-associated herpesvirus (KSHV) has been etiologically linked to several human cancers, including Kaposi’s sarcoma (KS), primary effusion lymphoma (PEL), and multicentric Castleman’s disease (MCD). However, recent studies suggest that KSHV infection may also be associated with the development of other diseases or increased risks, such as KSHV inflammatory cytokine syndrome (KICS), diabetes, malaria, heart disease, and other cancers. In this review, we summarize these findings from clinical observations, epidemiological studies or laboratory research, though more studies are needed in these emerging areas. We believe that this work will enhance our understanding of the molecular mechanisms underlying KSHV pathogenesis and contribute to improving treatments for related human diseases.

## 1. Introduction

Kaposi’s Sarcoma-associated herpesvirus (KSHV), also known as human herpesvirus 8 (HHV-8), is a γ-herpesvirus etiologically involved in Kaposi’s sarcoma (KS) and other related immunodeficiency disorders. KSHV is most prevalent in conditions such as acquired immunodeficiency syndrome (AIDS) due to co-infection with human immunodeficiency virus (HIV-1), as well as in KS, primary effusion lymphoma (PEL), and multicentric Castleman’s disease (MCD). Found in more than 95% of KS lesions, KSHV is a causative, though not the sole, agent in KS disease progression [1]. KSHV is a dsDNA encapsulated virus that infects host cells through attachment to cell receptors via viral glycoproteins. Binding and interaction of KSHV glycoproteins with integrins and other cellular receptors initiates intracellular signaling cascades that induce internalization, trafficking through the cytosol, and subsequent release of viral DNA into the nucleus, where it forms an episome that is attached to the host cell’s chromosomes [2]. KSHV has a biphasic lifecycle: a latent phase and a lytic phase. The latent phase depends on the Latency-Associated Nuclear Antigen (LANA) protein, which bridges the KSHV episome to the host chromosomes, allowing latent KSHV replication in daughter host cells. Upon activation by exogenous stimuli, KSHV transitions from the latent phase to the lytic phase [3]. KSHV reactivation from latency is initiated when various stimuli promote the expression of replication and transcription activator (RTA), the master lytic switch protein, which triggers a cascade of events that result in the modulation of various cellular pathways to support viral DNA synthesis and replication.

Kaposi’s sarcoma (KS) cases are categorized into four types: classic, which refers to individuals of Mediterranean, European Jewish, and Middle Eastern ancestry, where KS occurs without involvement of other disorders; endemic, which typically describes regions in Sub-Saharan Africa where KSHV is transmitted both vertically and horizontally; iatrogenic, which refers to KS cases caused by the transplantation of infected tissues into non-infected recipients or chronically immunocompromised or immunosuppressed patients, including post-transplant recipients; and the most prevalent category, AIDS-related KS. While the infection mechanisms of KSHV and its associated diseases—KS, PEL, and MCD—are well documented, recent studies have suggested probable connections between KSHV and other conditions, such as KSHV inflammatory cytokine syndrome (KICS), diabetes mellitus, osteosarcomas, prostate and cervical cancers, heart disease, as well as malaria and tuberculosis infections. This review aims to highlight these emerging associations and to consolidate the existing data into a comprehensive understanding of current KSHV interactions, advancing knowledge of this oncogenic herpesvirus.

## 2. KSHV Inflammatory Cytokine Syndrome (KICS)

KICS has been well documented since its discovery. The key characteristics of KICS include KSHV infection with an elevated viral load, increased cytokine production inducing inflammation, and elevated levels of IL-10, all of which are not typically seen in other diseases. KICS presents independently of other KS-associated diseases, such as MCD and PEL, but can occur in individuals with AIDS [4]. KICS generally presents with common symptoms, such as fever, sweats, fatigue, edema, breathing dysfunction, pain, and nausea with or without abdominal pain [5]. KICS can also occur in those who are receiving anti-retroviral therapies (ARTs) for HIV and therefore should be monitored for compounding disorders.

KICS has a high mortality rate, not only due to its cytokine- and immune-related effects but also because it often presents alongside other comorbidities, such as KS or AIDS. Currently, there is no specific, effective treatment for KICS, so multiple therapies are combined into a single treatment protocol. One such treatment involves rituximab, administered in tandem with liposomal doxorubicin or similar therapies. However, this combination can cause paradoxical issues [6]. Rituximab alone can trigger KS flares, so it is paired with liposomal doxorubicin to mitigate these side effects. Unfortunately, liposomal doxorubicin is metabolized in the liver. If a patient has liver dysfunction, liposomal doxorubicin becomes contraindicated, leaving only rituximab therapy, which may increase the frequency of KS flares. Liver-related issues were observed in approximately 35% of KS patients post-mortem [7], and more recent studies have identified hepatic Kaposi’s sarcoma (HKS) in ante-mortem examinations [8]. The numerical accuracy for KICS patients with hepatic involvement remains undistinguished, as KICS patients may be asymptomatic for liver dysfunction [8], potentially leading to undiagnosed liver issues, either for periods of time or even for life. Given that KSHV, the causal factor of KS, plays a role in KICS, it is crucial to explore alternative treatments that do not rely on hepatic metabolism, considering the associated liver dysfunction. Hepatic dysfunction disqualifies at least 35% of KICS patients from receiving rituximab and liposomal doxorubicin or similar treatments. As a result, these patients, unless they qualify for other combination therapies, are likely to experience more frequent KS flares due to rituximab and its pharmacokinetic interactions. As of now, KICS continues to have a high mortality rate due to these persistent treatment challenges.

KICS is entirely dependent on KSHV infection. While KSHV behaves similarly to other Herpesviridae in otherwise healthy hosts, remaining asymptomatic for extended periods [9], its impact on immunocompromised or immunosuppressed patients can be severe. The treatment of KICS is crucial, and understanding the underlying causative agent is even more important. Documenting cytokine signaling and cytotoxic mechanisms in KICS directly contributes to a better understanding of KSHV pathogenesis and its infectious pathways. Therefore, elucidating how KSHV modulates diseases and adjacent conditions is essential for global health and for developing potential therapies using KSHV as a delivery agent.

## 3. KSHV and Diabetes Mellitus

Diabetes mellitus (DM) is a widespread metabolic disorder, categorized either by the pancreas’s beta cells’ inability to produce insulin (Type 1 diabetes, T1D) or by the body’s inability to effectively use insulin (Type 2 diabetes, T2D), resulting in elevated glucose levels in the bloodstream. As of 2024, reports indicate that one in every nine people globally has diabetes, with approximately 90% of the 588.7 million diabetes cases being classified as Type 2 [10]. Although DM and its potential therapies are well studied, the immunopathology of how KSHV specifically affects diabetic patients remains sporadic. Given the large and rapidly growing global diabetic population, the intersection of KSHV and diabetes is becoming increasingly significant. Several studies have reported that patients with DM, especially T2D, have an elevated risk of KSHV infection [11,12,13], although a very recent study from Turkey reported no significant association between KSHV infection and diabetes [14]. These discrepancies are likely due to regional differences, methodological variations, or limited sample sizes.

**Figure 1:**
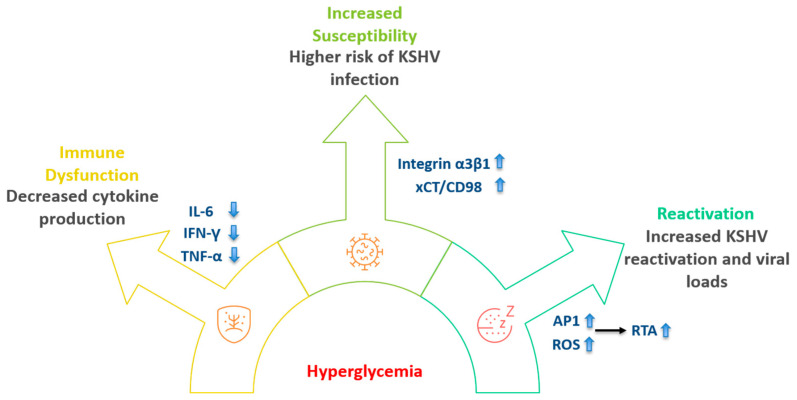
Hyperglycemia in diabetes patients may impair immune response to KSHV and facilitate virus infection and replication.

Diabetes has been documented as contributing to poorer immune function. Normally, when blood glucose levels rise, the hormone insulin is secreted by the pancreas’s islet beta cells, activating insulin receptors that interact with insulin receptor substrate 1/2 (IRS-1 and IRS-2) to promote the GLUT transporters, allowing glucose to enter cells [15]. Problems arise when either a lack of insulin prevents these processes or when cells become resistant to insulin. Insulin resistance is not solely due to a loss of insulin receptors but also involves complex dysregulation of other parts of the insulin signaling cascade, including intracellular mediators like c-Jun N-terminal kinases (JNKs) [16] and extracellular signals such as various adipokines [17]. Although acute hyperglycemia does not inherently lead to pro-inflammatory products, in diabetic patients, such episodes are more common and prolonged. Hyperglycemia-induced stress increases the presence of intracellular Reactive Oxygen Species (ROS) and activates the IKKβ substrate, which in turn activates the NF-κB pathway, promoting pro-inflammatory gene expression. This process has been reported to decrease several interleukin cytokines (including IL-2, IL-6, IL-10, and IL-1β), as well as Tumor Necrosis Factor-α (TNF-α) and other signaling molecules involved in immune function [15]. Diabetic individuals typically experience increased rates of infection due to these immune deficits, such as diabetic foot ulcers (DFUs) and slower wound healing, particularly in those with high HbA1c levels, a measure of average blood glucose over two to three months [18]. Higher HbA1c levels (above 5.7%) are generally associated with poorer health outcomes, reflecting chronic hyperglycemia. As previously mentioned, increased NF-κB activity plays a prominent role in KSHV pathogenesis. ROS is also critical to both the latent and lytic phases of the KSHV lifecycle [19], and increased ROS levels have been reported during chronic hyperglycemic episodes in T2D patients [15,18,19,20]. KSHV has been observed to require ROS for reactivation, using the oxidative pathway to mediate apoptosis and disperse virions [15]. While hyperglycemia may not directly affect the KSHV episome bridged by LANA, it appears to create favorable conditions for KSHV reactivation by increasing ROS and cellular stress. Thus, chronic hyperglycemia not only weakens immune function and increases initial susceptibility to KSHV but also worsens immune responses to KSHV infection and promotes KSHV reactivation following latent infection. A recent study found that activation of the transcription factor AP-1 by high glucose was crucial for KSHV lytic reactivation and that high glucose enhanced the susceptibility of various target cells to KSHV infection [12]. Particularly in endothelial and epithelial cells, the levels of specific cellular receptors for KSHV entry, including integrin α3β1 and xCT/CD98, were elevated under high-glucose conditions (Figure 1). As both T2D and T1D diagnoses continue to rise, the global population’s susceptibility to KSHV increases. While KSHV may not directly drive the immunological changes observed in DM, it benefits from the ideal conditions set by hyperglycemia. The inflammatory cytokine imbalances in DM worsen healing outcomes due to impaired immune responses, and we speculate that KSHV may exploit this environment to rapidly distribute virions during its reactivation. KSHV and DM exist in a symbiotic relationship, worsening patient outcomes, although more research is needed to clarify this relationship and the underlying mechanisms. Another important question is whether KSHV can directly infect pancreatic islet components, particularly beta cells, and the potential biological consequences of such an infection.

## 4. KSHV and Malaria

**Figure 2:**
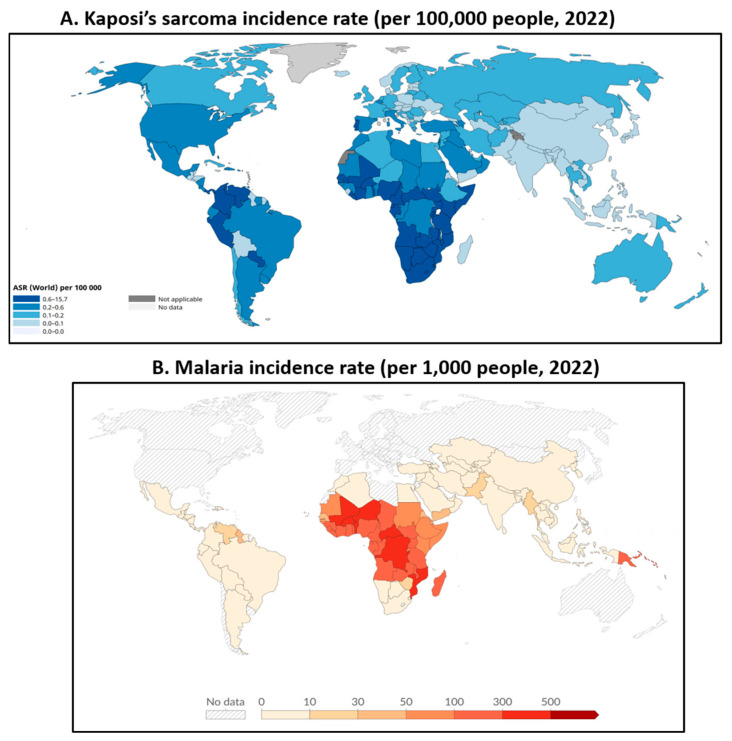
Global incidence rate and geographical distributions of confirmed cases of Kaposi’s sarcoma (**A**) and malaria (**B**). Data source: World Health Organization (Global Health Observatory), 2022.

Malaria is an endemic disease caused by strains of the single-celled protozoa *Plasmodium falciparum*, which is transmitted through the bites of female mosquitoes, including *Anopheles gambiae s.s.*, *Anopheles arabiensis*, and *Anopheles funestus*. While malaria has generally been concentrated in Africa, South America, and areas of the Middle East and the Mediterranean, recent climate changes have led to an increased geographical range of possible malaria infections, including Southern Asia, Southeastern and Eastern Asia, and North America [21]. With the expansion of malarial territories, the number of infectious co-conspirators has increased markedly. For example, a strong association between KS and malaria incidence through geographical overlap has been noticed, especially in many African countries (Figure 2).

As previously mentioned, KICS is an inflammatory response to KSHV infection in which up to 35% of patients, and perhaps more who are asymptomatic or undiagnosed, experience liver involvement and failure. Malaria can remove the possibility of hepatic-based metabolized treatments due to the incited liver dysfunction. Since *P. falciparum* relies on the host’s liver as its site of replication, hepatic damage can be very severe in malarial cases [22]. While severe liver damage is uncommon in typical malaria cases, it is more frequent in severe cases. The connection between KSHV, KICS, and malaria lies in liver involvement: many of the key drugs needed for KICS treatment are metabolized in the liver, and at least 35% of KICS patients are ineligible for these treatments due to hepatic insufficiency. Furthermore, since malaria’s primary target organ is the liver, where it ruptures hepatocytes for replication, the overlap of malaria, KSHV, and KICS may lead to significant yet often asymptomatic liver disease in regions where these diseases co-occur.

Studies linking malaria and KSHV infection have emerged over the past half-century. In two separate case–control studies in rural Uganda, associations were found between malaria infections and increased KSHV seropositivity [23,24]. These authors had previously reported that asymptomatic *Pf*(+) children had increased KSHV viral loads, a higher risk of KSHV, and increased KSHV antibody levels. Their continuing study reported that KSHV seropositivity increased sequentially from uninfected, to previously infected, to currently infected children. The assumption is that B cells are impacted by malaria infections, which trigger a potential switch from latency to lytic reactivation. Malaria has also been reported to reactivate other herpesviruses, such as herpes simplex virus (HSV), Epstein–Barr virus (EBV), and varicella zoster virus (VZV) [25,26,27,28,29].

## 5. KSHV and Reproductive Cancers

Reproductive cancers, such as cervical cancer and prostate cancer, are leading causes of mortality among women, men, and intersex individuals. According to the International Agency for Research on Cancer’s 2022 annual report, prostate cancer caused 397,430 deaths, while cervical cancer caused 348,874 deaths [30].

### 5.1. Cervical Cancer

Both KSHV and human papillomavirus (HPV) are double-stranded DNA (dsDNA) viruses with oncogenic properties. HPV is a known determinant for cervical cancer, although the virus can also cause benign and precancerous lesions in various areas not related to the host’s reproductive or sexually active organs. Like KSHV, HPV infects areas of contact, particularly mucosal membranes, and has been detected in the oral cavity, on the epidermis, in the cervical opening, and even on the larynx and vestibular folds in some cases. Besides similar transmission routes, KSHV and HPV share other characteristics: both possess latent and lytic life phases, and both form episomes that reside in the nucleus for viral replication [3,31]. Chronic, untreated HPV infections in the cervix are the etiological cause of 95% of cervical cancers [32]. Unlike KSHV, HPV displays an evident pathophysiological pathway. However, research examining the relationship between these two viruses remains scarce. One study about KSHV infection in sex workers and women from the general population in Spain reported that KSHV DNA was detected in 2% of the cervical samples of prostitutes and in 1% of the cervical samples of women in the general population [33]. Another study reported oral co-infection of HPV and KSHV among HIV-infected individuals in Italy, especially men who have sex with men [34]. Our group reported for the first time that the HPV16-integrated cervical cancer cell line SiHa is susceptible to KSHV latent infection [35]. Array analysis indicated that KSHV co-infection induced the production of some inflammatory cytokines/chemokines, such as IL-6, MIF, CXCL1, and CCL5, in SiHa cells. More research is required to understand the potential contribution of KSHV co-infection to cervical cancer development. Interestingly, our previous data indicated that KSHV infection significantly reduced both E6 and E7 expression in HPV from SiHa cells. Furthermore, we found that LANA and vFLIP, two major KSHV-encoded latent proteins, were responsible for the downregulation of E6 and E7 expression in SiHa cells [35]. In contrast, another group found that the KSHV lytic protein RTA could bind to various HPV16 genomic regions and induce a significant upregulation of E7 transcription [36], suggesting that KSHV latent and lytic proteins may differently regulate HPV oncogenic protein expression. However, the exact impact of HPV on KSHV gene expression remains unclear.

### 5.2. Prostate Cancer

As many as one in six men possess a risk of a prostate cancer diagnoses [37], with risk increasing with age. However, KSHV may play a role with prostate cancer regardless of its oncogenic requirement in disease progression. In the 1990s, there was some discourse over whether KSHV was present in prostate tissue, with some studies stating that KSHV did not have a normal latency progression in prostate cancer development [38], while others argued for KSHV’s ubiquitous presence in the male prostate and semen [39,40]. Elevated seroprevalence of KSHV has been found among men with prostate cancer, especially those from Tobago [41,42]. A similar cohort study also found a significant association between KSHV infection and increased levels of serum PSA, a biomarker for prostate cancer [43]. In 2016, the intrinsic role of oxidative stress (OS) in prostate cancer was examined [44]. OS was determined to play an integral role in the progression of prostate cancer, and as previously mentioned, KSHV relies on increased OS for moderation of latency and lytic life stages. Not only does age increase the risk of prostate cancer, but age also increases OS production, which can induce reactivation from latent KSHV infections. While KSHV may not be the direct perpetrator of prostate cancer [45,46], OS is directly attributed to high levels of ROS, which are both tumorigenic and favorable for KSHV lytic reactivation [47]. Although targeting ROS function is not a new cancer treatment strategy, adapting such treatments could be key to limiting KSHV reactivation. An interesting study reported that KSHV infection of androgen-sensitive prostate cancer cells confers the ability for androgen-independent growth. Mechanistically, KSHV infection bypassed androgen receptor (AR) signaling by promoting enhancer of zeste homolog 2 (EZH2)-mediated epigenetic silencing of tumor-suppressor genes, including MSMB and DAB2IP [48]. Additionally, KSHV infection triggered epithelial-to-mesenchymal transition (EMT) [49], which may further promote prostate cancer development.

## 6. KSHV and Heart Disease

Dilated cardiomyopathy (DCM) is the leading cause of heart transplantation. The underlying etiologies of DCM are varied and include genetic mutations, infectious agents (particularly viruses), toxins like alcohol, chemotherapeutic agents, as well as autoimmune and systemic disorders [50]. Biopsy-proven viral myocarditis has been reported in 9–16% of adult patients with DCM, and up to 30% of these patients with viral myocarditis might progress to DCM [51]. Interestingly, one recent study found that KSHV infection is closely related to DCM in some patients [52]. In their study, increased KSHV seropositivity and quantitative titers were found in patients with DCM compared with the non-DCM group. The risk of the individual endpoints of death from cardiovascular causes or heart transplantation was increased among DCM patients with KSHV DNA seropositivity during follow-up. In heart tissues, the KSHV DNA load was also increased in the hearts of patients with DCM in comparison with healthy donors. Moreover, one of the KSHV microRNAs, kshv-miR-K12-1-5p, was detected in both the endothelium and cardiomyocytes of some DCM patients. The overexpression of kshv-miR-K12-1-5p aggravated known cardiotropic viruses-induced cardiac dysfunction and inflammatory infiltration [52]. Given the increased detection of KSHV in clinical settings with improved technologies, we speculate that KSHV will be linked to a broader range of heart diseases. However, it should be noted that there is currently no direct evidence of KSHV infection in human cardiomyocytes. If such infection occurs, it remains unclear what specific pathological features of myocardial dysfunction KSHV might cause. These questions will need to be addressed in future studies.

## 7. KSHV and Osteosarcoma

Osteosarcoma is the most common malignant tumor of bone, with an incidence of approximately three cases per million annually worldwide, predominately affecting adolescents and young adults [53,54]. A viral etiology for osteosarcoma has been suggested based on some animal studies [55,56]. However, this viral etiology has never been proven by the identification of any virus that is authentically associated with human osteosarcoma. The Uyghur ethnic population in Xinjiang, China, has an unusually high prevalence of KSHV infection and an elevated incidence of osteosarcoma. Chen et al. recently reported that KSHV infection is a risk factor for osteosarcoma and that KSHV is associated with some osteosarcomas [57]. They found that KSHV prevalence was significantly elevated in Uyghur osteosarcoma patients versus the general Uyghur population. The KSHV DNA genome and viral major latent protein LANA were detected in most osteosarcoma tumor cells. Gene expression profiling analysis showed that KSHV-positive osteosarcoma represents a distinct subtype of osteosarcomas with viral gene-activated signaling pathways important for tumor development [57]. It would be intriguing to determine whether KSHV-associated osteosarcoma is unique to the Xinjiang Uyghur population or if it also occurs in other populations with a high prevalence of KSHV, such as those in East Africa.

## 8. Conclusions

Unlike KS, PEL, and MCD, KSHV has not been linked etiologically to most of the human diseases we discussed above, except for KICS, which represents a newly characterized syndrome caused by KSHV infection. Currently, the involvement of KSHV in these diseases is supported by three types of evidence: (I) in vitro models (e.g., cervical cancer, prostate cancer); (II) epidemiological data (e.g., diabetes mellitus, malaria, heart disease); and (III) the presence of KSHV in relevant cells (e.g., osteosarcoma). However, in some cases, the detection of KSHV may be incidental rather than causal. Nevertheless, it is highly likely that KSHV infection or co-infection may act as a co-factor, influencing the development, pathogenesis, or risk of certain diseases. Therefore, further research is needed to clarify these roles, particularly through mechanistic studies, which may open new directions in KSHV research. Moreover, such studies will significantly increase our understanding of the molecular mechanisms underlying KSHV pathogenesis and help improve treatments for related human diseases.

## Data Availability

All the data shown in this paper are available from the corresponding authors upon reasonable request.

## Abbreviations

KSHV	Kaposi’s sarcoma-associated herpesvirus
KICS	KSHV inflammatory cytokine syndrome
KS	Kaposi’s sarcoma
PEL	Primary effusion lymphoma
MCD	Multicentric Castleman’s disease
ART	Anti-retroviral therapies
HHV-8	Human herpesvirus 8
LANA vFLIP	Latency-associated nuclear antigen Viral FLICE-inhibitory protein
*Pf*	*Plasmodium falciparum*
DM	Diabetes mellitus
OS	Oxidative stress
ROS	Reactive oxygen species
PSA	Prostate-specific antigen
DCM	Dilated cardiomyopathy
EMT	Epithelial-to-mesenchymal transition
